# Hemoglobins Likely Function as Peroxidase in Blood Clam* Tegillarca granosa* Hemocytes

**DOI:** 10.1155/2017/7125084

**Published:** 2017-01-15

**Authors:** Sufang Wang, Xiaopei Yu, Zhihua Lin, Shunqin Zhang, Liangyi Xue, Qinggang Xue, Yongbo Bao

**Affiliations:** ^1^School of Marine Sciences, Ningbo University, Ningbo 315211, China; ^2^Zhejiang Key Laboratory of Aquatic Germplasm Resources, Zhejiang Wanli University, Ningbo 315100, China

## Abstract

Hemoglobins are a group of respiratory proteins principally functioning in transport of oxygen and carbon dioxide in red blood cells of all vertebrates and some invertebrates. The blood clam* T. granosa* is one of the few invertebrates that have hemoglobin-containing red hemocytes. In the present research, the peroxidase activity of* T. granosa* hemoglobins (Tg-Hbs) was characterized and the associated mechanism of action was deciphered via structural comparison with other known peroxidases. We detected that purified Tg-Hbs catalyzed the oxidation of phenolic compounds in the presence of exogenous H_2_O_2_. Tg-Hbs peroxidase activity reached the maximum at pH 5 and 35°C and was inhibited by Fe^2+^, Cu^2+^, SDS, urea, and sodium azide. Tg-Hbs shared few similarities in amino acid sequence and overall structural characteristics with known peroxidases. However, the predicted structure at their heme pocket was highly similar to that of horseradish peroxidase (HRP) and myeloperoxidase (MPO). This research represented the first systemic characterization of hemoglobin as a peroxidase.

## 1. Introduction

Hemoglobins (Hbs) are iron-containing respiratory proteins existing in red blood cells of all vertebrates and some invertebrates [1]. The principal function of hemoglobins is to transport oxygen and carbon dioxide in the circulation system of animals. Studies have revealed that hemoglobins play roles in host immunity and antioxidant and molting regulation [2]. In addition, there has been evidence showing the presence of “pseudoperoxidase” or peroxidase-like activities in hemoglobins [3–8]. It was proposed that hemoglobins can cause oxidative injuries through production of oxidative radicals [9, 10]. At the same time, some researchers speculated that hemoglobin peroxidase-like activity may promote oxygen-dependent microbicidal action by catalyzing reactions to produce superoxide ions including some toxic derivatives such as hydroxyl radicals and hypohalous acid [7]. However, these enzyme activities of hemoglobins have been rarely characterized.

The blood clam,* T. granosa*, is a major fishery and aquacultural bivalve mollusk living on the east coast of China and Southeast Asia. The clam belongs to the family Arcidae, one of a few invertebrate groups that have hemoglobin-containing red hemocytes in the hemolymph. It has been estimated that more than 90% of total* T. granosa* hemocyte proteins are hemoglobins [11, 12].* T. granosa* hemoglobin (Tg-Hb) exists in two forms: Tg-HbI and Tg-HbII. Tg-HbI is a homogenous dimer consisting of identical subunits, whereas Tg-HbII is a heterogeneous tetramer formed by two types of subunits that differ from each other and from Tg-HbI subunit in molecular weight and isoelectric point [13]. Research results have shown that Tg-HbI and Tg-HbII both have antibacterial activities [14]. The mechanisms underlying the activities, however, remain to be investigated.

In a previous research, Tg-Hbs have been detected to have peroxidase activity [14]. The objectives of the present research were to determine the enzymatic characteristics of the two Tg-Hbs in catalyzing oxidative reactions and the related mechanism. The results should help in better assessing hemoglobin functions in host defense of bivalve mollusks in the Arcidae family.

## 2. Materials and Methods

### 2.1. Materials

Tg-Hbs were purified from* T. granosa* hemocytes as reported previously [15]. Guaiacol (GA), L-dihydroxyphenylalanine (L-DOPA), lipopolysaccharide (LPS), and lipoteichoic acid (LTA) were purchased from Sigma-Aldrich. All other chemicals were products of the highest analytical grade.

### 2.2. Peroxidase Activity Assay

Peroxidase activity was measured by the method previously described [16] with some modifications. The method is based on the generation of tetraguaiacol from guaiacol by peroxidase in the presence of H_2_O_2_. Measurements were performed by mixing 1 ml of substrate solution containing 4 mM guaiacol, 2 mM H_2_O_2_, and 50 mM sodium phosphate buffer, with pH 7.0 as substrate buffer with 10 *µ*l of Tg-Hb at 3 mg/L in substrate buffer. After brief shaking to mix thoroughly, the mixture was measured continuously for 2 min at room temperature for the absorbance at 470 nm using a Shimadzu UV-1800 spectrophotometer connected with a recorder. As the reaction consists of the formation of one molecule of tetraguaiacol and 4 molecules of H_2_O from 4 molecules of guaiacol and 2 molecules of H_2_O_2_, the enzyme activity was calculated from Δ*A*_470_/min according to the following formula: *ε*(tetraguaiacol)_470_ = 26600 M^−1 ^cm^−1^.

### 2.3. Peroxidase Property Characterization

The optimal temperature for Tg-Hb peroxidase activity was determined by measuring the activities at temperatures between 10°C and 60°C in the buffer described earlier. The optimal pH was determined by measuring the activities in citrate or Tris-HCl buffers at different pH values ranging from pH 2 to pH 10 at 25°C. Kinetics assays were carried out to determine Michaelis–Menten constants.

To test the effects of SDS, urea, guanidine hydrochloride, lipopolysaccharide (LPS), lipoteichoic acid (LTA), NaN_3_, and metal ions on Tg-Hb peroxidase activity, related materials dissolved in 50 mM acetic acid buffer (pH 5.0) at different concentrations were incubated with purified Tg-Hbs for 2 h at 25°C, and peroxidase activities were then measured. All measurements were done in triplicate.

### 2.4. Sequence Based Biochemical Property Prediction and Comparison

The amino acid sequences of Tg-Hb, MPO, and HRP were retrieved from the NCBI GenBank (https://www.ncbi.nlm.nih.gov/). Basic biochemical properties of related proteins were predicted by the program ProtParam (http://web.expasy.org/protparam). Multiple sequence alignments were done using the program Clustal X and colored using the program ESPript (http://espript.ibcp.fr/ESPript/cgi-bin/ESPript.cgi).

### 2.5. Tertiary Structure Prediction and Activity Site Analysis

Three-dimensional structures of Tg-Hbs were modeled using the program SWISS-MODEL (https://swissmodel.expasy.org/) with HbI (PDB 3g53) and HbII (PDB 4hrr) from* Scapharca inaequivalvis* as templates. The heme and the related key amino acid residues of the analyzed proteins were displayed with Chimera (version 1.11.2).

## 3. Results

### 3.1. Peroxidase Activity of Tg-Hb

**Figure 1:**
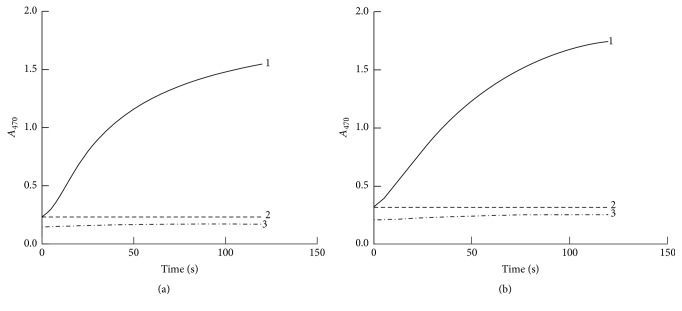
The peroxidase activity of Tg-Hb. (a) Tg-HbI, (b) Tg-HbII; 1: Tg-Hb + guaiacol + H_2_O_2_, 2: Tg-Hb + H_2_O_2_, and 3: Tg-Hb + guaiacol.

The absorbance of reaction tubes containing substrate and either Tg-HbI (Figure 1(a)) or Tg-HbII (Figure 1(b)) increased with incubation time. In contrast, the absorbance of reaction tubes containing Tg-Hbs plus incomplete substrate solutions without either H_2_O_2_ or guaiacol did not change with incubation time (Figures 1(a) and 1(b)).

### 3.2. Temperature and pH Optima

**Figure 2:**
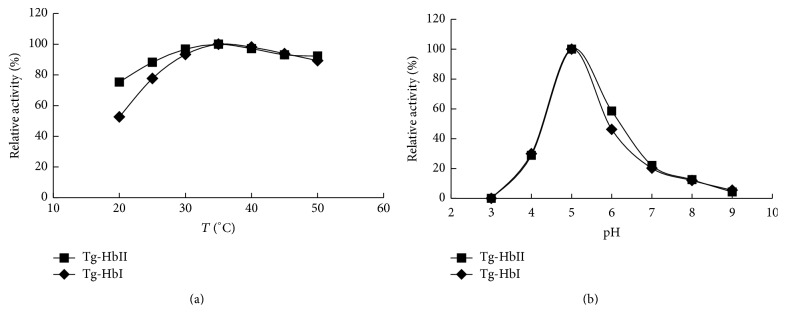
Effect of temperature and pH on Tg-Hb activity.

The peroxidase activities of purified Tg-Hbs measured at various temperatures and pH values were shown in Figure 2. The relative peroxidase activity of both Tg-Hbs increased from 20°C to 35°C and then decreased gradually. At 50°C, the relative activity retained about 80% of the maximum (Figure 2(a)). At pH 3, the peroxidase activity of Tg-Hbs was not detected. The relative activity then increased to 30% at pH 4 and reached the maximum at pH 5 and then decreased as pH increased. At pH 9, both Tg-Hbs did not have detectable peroxidase activity (Figure 2(b)).

### 3.3. Michaelis–Menten Constants and Substrate Specificity

**Figure 3:**
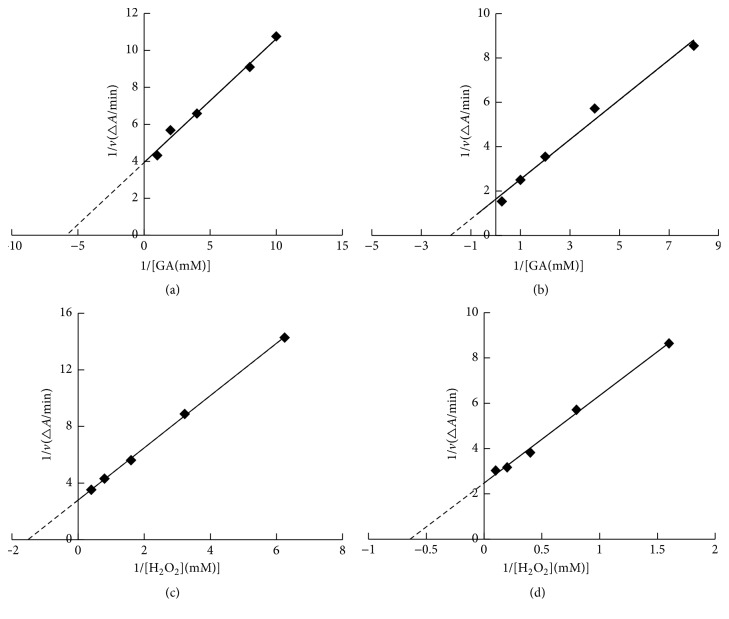
Lineweaver–Burk plot for the Michaelis–Menten constant. (a, c) Tg-HbI; (b, d) Tg-HbII.

Michaelis–Menten constants of Tg-Hbs catalyzing guaiacol were calculated from the Lineweaver–Burk plot (Figure 3). The calculated constants were *K*_  m_^GA^ = 0.15 and *K*_m_^H_2_O_2_^ = 0.54 for Tg-HbI and *K*_m_^GA^ = 0.39 and *K*_m_^H_2_O_2_^ = 0.40 for Tg-HbII. The catalysis of oxidization of catechol, hydroquinone, phenol, and dopamine was also measured and the related Michaelis–Menten constants were shown in Table 1.

### 3.4. Effects of Chemicals on Tg-Hb Peroxidase Activity

**Figure 4:**
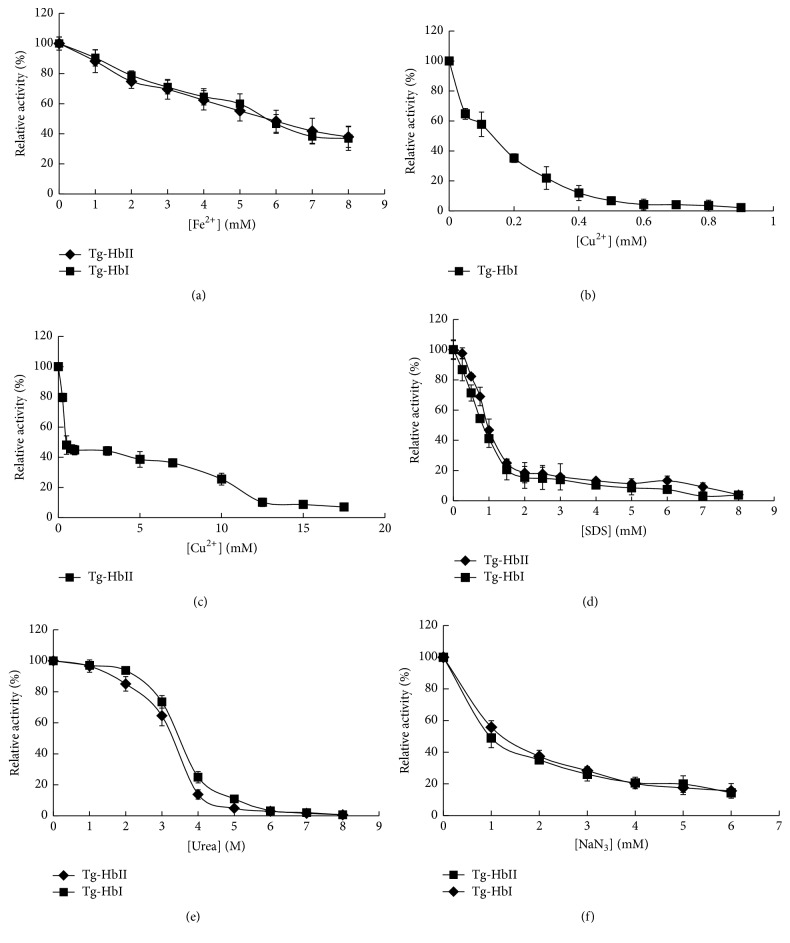
The effect of chemicals on Tg-Hb peroxidase activity.

The peroxidase activity of Tg-HbI and Tg-HbII decreased with the presence of Fe^2+^ at incremental concentrations from 1 mM to 8 mM. When 8 mM Fe^2+^ was added in the reaction, only 40% of the original enzyme activity was retained (Figure 4(a)). The activity also decreased with the addition of Cu^2+^ in the reaction. Tg-HbI peroxidase activity was almost completely abolished when Cu^2+^ concentration reached 0.5 mM (Figure 4(b)). Although Tg-HbII peroxidase activity decreased sharply with Cu^2+^ increase at the low concentration range, it tolerated relatively higher Cu^2+^ concentrations and retained more than 20% maximal activity in the presence of 10 mM Cu^2+^ (Figure 4(c)). Metal ions of Mn^2+^, Mg^2+^, Zn^2+^, and Pb^2+^ were not detected to impact on Tg-Hb peroxidase activity.

Tg-Hb peroxidase activity decreased drastically with the addition of SDS at increasing concentrations (Figure 4(d)). The activity retained 20% of the maximum and almost completely lost with presence of SDS at 1.5 mM and 8 mM, respectively. The peroxidase activity of both Tg-Hbs was retained at urea concentration up to 2 M and decreased with the elevation of urea concentration. When urea concentration reached 6 M, no peroxidase activity was detected in both proteins (Figure 4(e)).

Tg-Hb peroxidase activity was also inhibited by NaN_3_ in a dose-dependent manner (Figure 4(f)). The activity decreased by 50% and 80% when NaN_3_ was added to 1 mM and 5 mM, respectively.

### 3.5. Effects of LPS and LTA on Tg-Hb Peroxidase Activity

**Figure 5:**
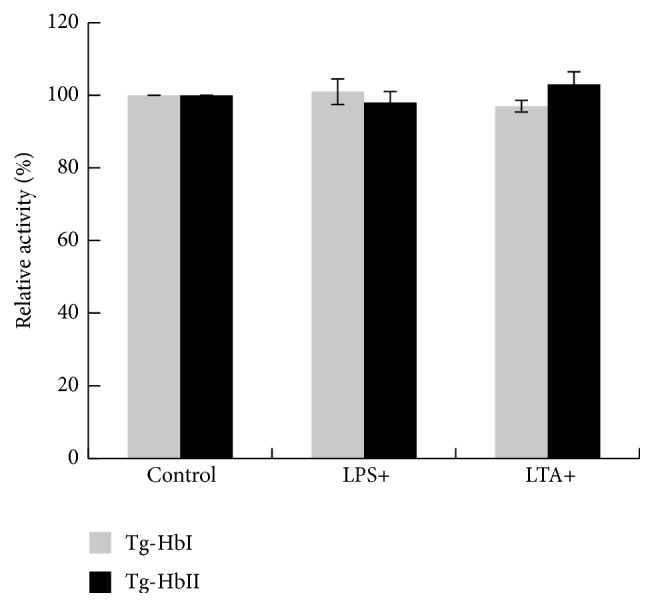
The effect of LPS and LTA on Tg-Hb peroxidase activity.

LPS and LTA at the concentration of 100 *μ*g/ml did not show effects on the peroxidase activity of the two Tg-Hbs (Figure 5).

### 3.6. Biochemical Comparison between Tg-Hbs and the Representative Peroxidases

The predicted biochemical characteristics of the three types of subunit (Tg-Hbi that forms the dimer Tg-HbI and Tg-Hbii*α* and Tg-Hbii*β* that form the tetramer Tg-HbII) were compared with those of horse radish peroxidase (HRP), a representative of plant peroxidase, and myeloperoxidase (MPO), a representative peroxidase of vertebrates. As shown in Table 2, the subunits differed from each other in molecular weight, isoelectric point (pI), cysteine residues number, and total amino acids number.

### 3.7. Sequence Comparison between Tg-Hb Subunits and Representative Peroxidases

**Figure 6:**
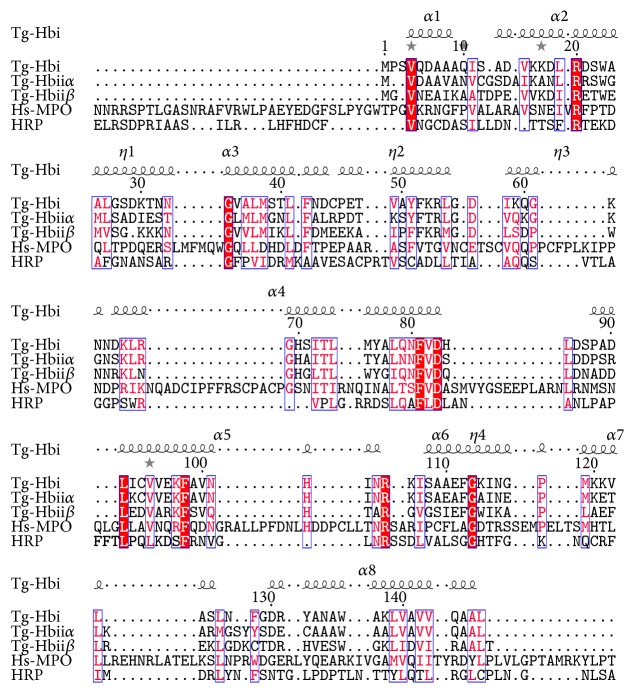
Amino acid sequence alignment of four proteins. Same amino acid residues are shaded in red, similar amino acids are shaded in box, and *α*-helices are shown as *α*1 to *α*8.

The amino acid sequence of the three Tg-Hb subunits was aligned with that of HRP and the MPO heavy chain (Figure 6). The similarity between these polypeptides at the primary structural level was low. The MPO light chain was not included in the alignment because it does not contain heme and the overall sequence similarity is even lower.

### 3.8. Predicted Protein and Heme Pocket Structures

**Figure 7:**
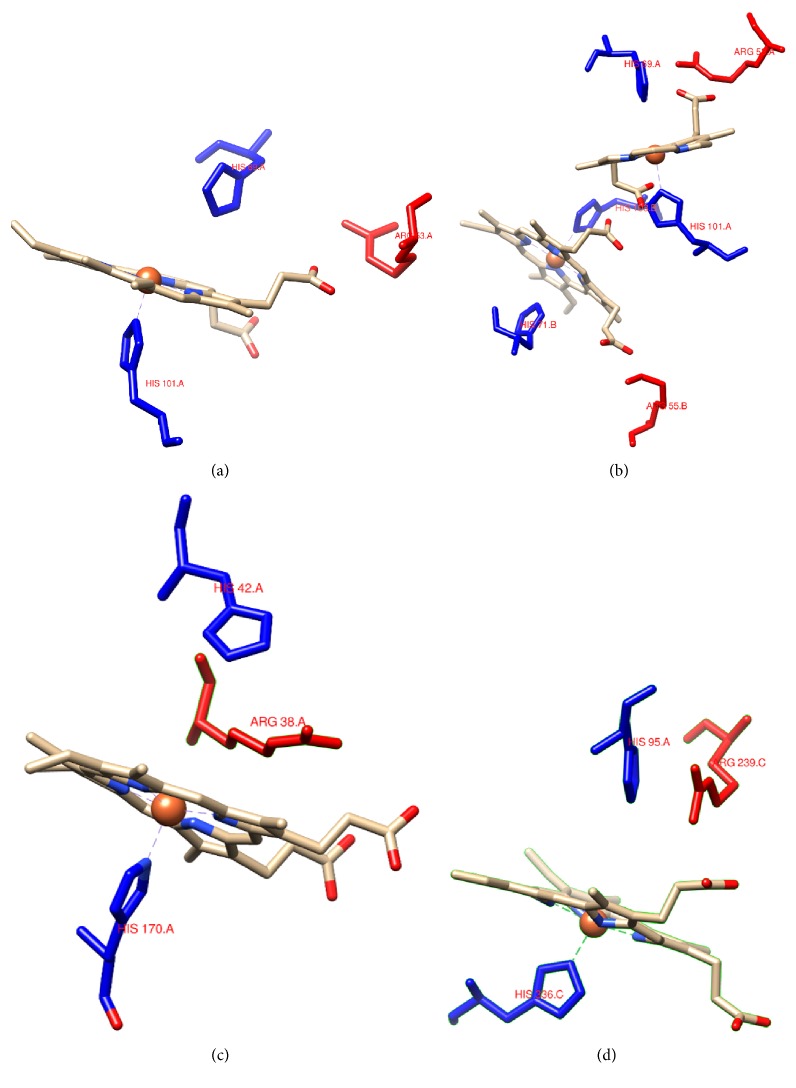
The heme and near key residues of Si-Hb I (a), Si-Hb II (b), HRP (c), and MPO (d).

Sequence alignment indicated that Tg-Hbi shared 81.51% sequence identity with the* S. inaequivalvis* hemoglobin (Si-Hb) subunit 3g53 in the PDB, and Tg-Hbii*α* and Tg-Hbii*β* shared 91.23% and 36.7% sequence identity, respectively, with the Si-Hb subunit 4hrr in the PDB. The 3D structures of the three Tg-Hb subunits were modeled using the SWISS-MODEL program with Si-Hb subunits as templates. Tg-Hb subunits shared similarities with related Si-Hb subunits in secondary structural elements including *α*-helices, random coil, and *β*-sheets and tertiary structure. The predicted heme pocket of Tg-Hb subunits was particularly similar to that of Si-Hb in amino acid sequence and tertiary structure. Therefore, the heme structure and key residues in the pocket of the Si-Hb subunits were compared with those of HRP and MPO (Figure 7). All the compared structures contained a type b heme, of which the substrate oxidation site was formed by a region that included the heme methyl C18 and meso protons of heme C20. In addition, the positions of the proximal His and distal His and the Arg residues that are key to the functionality of the heme pocket were also conserved (Figure 7). Tg-Hb subunits show little similarity with HRP and MPO at the tertiary level.

## 4. Discussion

Hemoglobins represent a group of oxygen transport proteins present in red blood cells of all vertebrates and the red hemocytes of some invertebrates. There are increasing research results that suggest immune functions of these proteins. The blood clam* T. granosa* is one of the few invertebrates that have hemoglobins. Tg-Hbs have been studied for the potential function in host immunity. Findings in the present research indicated that Tg-Hbs may function as peroxidase in the clam's hemocytes.

Purified Tg-Hbs catalyzed the oxidation of several phenol compounds in the presence of H_2_O_2_, with high affinity to guaiacol and H_2_O_2_ as indicated by the related* K*m values. Spectrophotometrically monitoring the conversion of the colorless guaiacol to the dark brown tetraguaiacol is a standard technique to measure peroxidase activity [17]. Although phenoloxidases also catalyze phenol oxidation, the catalysis does not require exogenous H_2_O_2_ [18]. In the present research, the oxidation activity of purified Tg-Hbs was not detected when H_2_O_2_ was absent in the reactions. In addition, Tg-Hbs showed maximal peroxidase activity at pH 5.0 and the activity was inhibited by NaN_3_. These enzymatic characteristics conformed to those of HRP and MPO [19]. Therefore, Tg-Hbs possibly possess peroxidase activity.

Several factors affected the peroxidase activity of Tg-Hbs. In addition to temperature, pH, and NaN_3_, some metal ions and protein denaturants affected the activity. A possible reason for Fe^2+^ inhibiting Tg-Hb peroxidase activity is the consumption of H_2_O_2_ by the oxidization of the ferrous ions as H_2_O_2_ is essential for the peroxidase catalysis. Differences in sensitivity and response pattern to Cu^2+^ were observed between Tg-HbI and Tg-HbII which suggested that a more complex mechanism was likely involved in the response of Tg-Hbs to the metal ion. For the protein denaturants, SDS disrupts hydrophobic interactions in protein molecules, while urea interacts with amide groups and peptides to destabilize protein structures [20]. These interactions can change the conformations of Tg-Hbs, in particular the active center (i.e., the heme pocket), resulting in the loss of peroxidase activity. It has been reported that sodium azide is a proven inhibitor of peroxidases including MPO and HRP, and the inactivation involves the binding of sodium azide to the heme, thus preventing substrates from oxidization [20, 21].

It is worth noting that the two Tg-Hbs showed some differences in peroxidase activities. For example, they have different* K*m values for the tested substrates, suggesting differences in substrate specificities. They also significantly differed in tolerating the inhibition by Cu^2+^. In addition, Tg-HbI and Tg-HbII are different in subunit types and subunit number [14]. Future studies should be done to assess whether the differences in enzymatic properties are determined at the subunit level or at the quaternary structure level.

HRP and MPO are hemoproteins and both have a hydrophobic heme pocket that contains a type b pentacoordinated heme with proximal His and distal His, being critical for the peroxidase activity. The open site of the heme can bind oxygen (O_2_), nitric oxide (NO), carbon monoxide (CO), or H_2_O_2_ [19, 22]. When bound to the heme, H_2_O_2_ oxidizes the Fe (III) state heme to generate a higher oxidative state intermediate comprising Fe (IV) oxoferryl center and a porphyrin-based cation radical [23]. The oxidative intermediate then gives rise to adducts such as C18-hydroxymethyl and C20-meso-phenyl heme derivatives, which can further oxidize the bound substrate [24]. The distal His residue functions as a receptor of the H_2_O_2_ proton and to bind and stabilize ligands and aromatic substrates together with the Arg residue in the heme pocket [22, 24, 25]. Our results in comparison of the heme pocket suggested that the structural elements essential for the peroxidase activity are highly similar between Si-Hb and the two known peroxidases. As the Tg-Hb heme pocket is identical to the Si-Hb heme pocket, it is reasonable to predict that the mechanism of action underlying the detected Tg-Hb peroxidase activity should be the same as the other known peroxidases.

Xu et al. reported that the* Scapharca kagoshimensis* hemoglobins have phenoloxidase activity [26]. We did not detect any catalysis activity in purified Tg-Hbs when measured without H_2_O_2_; thus the phenoloxidase activity could not be confirmed in the* T. granosa* hemoglobins. It should be mentioned that, unlike hemoglobin and other hemoproteins, hemocyanin and phenoloxidase have copper as prosthetic for their enzyme activity. In addition, studies on hemocyanin have revealed that treatments with SDS, proteolytic enzymes, PAMPS, and pathogen invasion can convert the invertebrate oxygen transport protein to have phenoloxidase activity [27–30]. Jiang et al., for example, reported that LPS, LTA, and bacteria proteinase could activate the phenoloxidase activity of hemocyanin [7]. However, LPS and LTA did not show any effects on Tg-Hb enzyme activity in the present study. Further research is needed to draw the conclusion whether Tg-Hbs have other enzyme activities.

There is evidence for the involvement of peroxidases in host defense [31–34]. For example, MPO and lactoperoxidase (LPO) have been well studied for their antibacterial activity [35–37]. It has been reported that MPO plays an important role in the microbicidal activity of phagocytes. When neutrophils engulf pathogens, they also produce H_2_O_2_ along with the respiratory burst. With the presence of H_2_O_2_, MPO oxidizes halide (particularly chloride) ion to form highly reactive halide-derived oxidants [37–39].* T. granosa* red hemocytes appeared to phagocytose particles [40]. Given the hemoglobin's extreme abundance in red hemocytes, it is plausible to speculate that Tg-Hbs may function in the phagocytosis of the clam hemocytes in the same way as MPO in neutrophils. Studies observing peroxidase activity change in hemocytes during pathogen phagocytosis should provide further information about the function of Tg-Hbs in* T. granosa* host immunity.

## Figures and Tables

**Table 1:** Michaelis–Menten constants of Tg-Hb.

Phenols	Tg-HbI		Tg-HbII	
	*K*_m_^p^	*K*_ m_^H_2_O_2_^	*K*_m_^p^	*K*_ m_^H_2_O_2_^
Guaiacol	0.15	0.54	0.39	0.40
Pyrocatechol	0.097	7.28	0.087	0.37
*L*-DOPA	1.44	1.97	2.65	1.25
Phenol	1378.55	1.07	4.49	1.81
Hydroquinone	108.25	1.62	5.77	10.09

Note: *K*_m_^p^: Michaelis–Menten constants for phenols.

**Table 2:** Basic biochemical properties of Tg-Hb, MPO, and HRP.

Peptides	Accession	Species	Molecular weight	pI	Cys	Amino acids number
Tg-Hbi	HQ149305	*T. granosa*	16037.4	8.89	2	147
Tg-Hbii*α*	HQ729976	*T. granosa*	16231.7	8.81	3	150
Tg-Hbii*β*	HQ149306	*T. granosa*	17232.7	5.66	1	152
HRP	1KZM_A	*Armoracia rusticana*	33841.1	5.47	6	308
MPO H	1MHL_C	*Homo sapiens*	53164.5	9.48	12	466
MPO L	1MHL_A	*Homo sapiens*	12318.9	5.77	2	108

Note: Tg-Hbi: Tg-HbI subunit; Tg-Hbii*α*: Tg-HbII subunit *α*; Tg-Hbii*β*: Tg-HbII subunit *β*; MPO H: MPO heavy chain; MPO L: MPO light chain.
